# The combinatorial effect of age and biological sex on alloimmunity and transplantation outcome

**DOI:** 10.3389/frtra.2023.1325232

**Published:** 2024-01-09

**Authors:** Friederike Martin, Yao Xiao, Vanessa Welten, Keita Nakamori, Merih Gizlenci, Hao Zhou, Stefan G. Tullius

**Affiliations:** ^1^ Division of Transplant Surgery, Department of Surgery, Brigham and Women’s Hospital, Harvard Medical School, Boston, MA, United States; ^2^ Department of Surgery, Campus Charité Mitte|Campus Virchow-Klinikum, Charité—Universitätsmedizin Berlin, Berlin, Germany; ^3^ Department of Surgery, Brigham and Women’s Hospital, Harvard Medical School, Boston, MA, United States; ^4^ Department of Urology, Osaka Medical and Pharmaceutical University, Osaka, Japan

**Keywords:** age, sex, sex hormones, immunosenescence, alloimmunity and transplantation

## Abstract

Both age and biological sex affect transplantation outcomes. We have recently shown in a large volume clinical analysis utilizing the SRTR data that graft survival is inferior in young female kidney transplant recipients. In this multi-factorial analysis, older female recipients presented with a trend towards improved transplant outcomes compared to both young female recipients and male recipients of any age. Those data supported by reports of those of others suggest that sex and age impact alloimmune responses both, individually and synergistically. Biological sex and hormone levels change throughout a lifetime with recognized effects on longevity in addition to an impact on the development and course of several disease preconditions. Detailed mechanisms of those sex and age-specific aspects have thus far been studied outside of transplantation. Effects on alloimmunity are largely unknown. Moreover, the combinatorial impact that both, biological sex and age have on transplant outcomes is not understood. Here, we summarize available data that analyze how age in combination with biological sex may shape alloimmune responses and affect transplant outcomes.

## Introduction

Solid organ transplantation (SOT) is the only effective treatment for patients with end-stage organ failure. Several recipient and donor-related factors including age and biological sex have been identified as risk factors affecting transplantation outcomes (1, 2).

Biological sex has been recognized in many disease processes as impactful. Effects on alloimmunity and transplantation outcomes have not been studied extensively. In recent years, evidence has grown that recipient sex in addition to donor-recipient sex mismatches affect transplantation (3–7). Possible explanations include differences in sex-hormone levels, genetic disparities, epigenetic variations, differences in the microbiome as well as organ-specific size discrepancies (6).

Existing clinical data suggest recipient age to be a relevant confounder for sex-related outcome differences after SOT (3, 8, 9). With an overall aging population and increasing life expectancies worldwide, the number of elderly patients necessitating and undergoing SOT has increased. Based on recent Organ Procurement & Transplantation Network (OPTN) data (2022), 40.2% and 22.8% of all organ recipients in the US have been 50–64 years and ≥65 years, respectively. In contrast, in 2000, 36.2% were 50–64 years and only 7.8% ≥65 years (data retrieved September 21, 2023). We have been able to show in a recent analysis of the Scientific Registry of Transplant Recipients (SRTR) database that graft survival is inferior in young female recipients. In contrast, older female recipients have shown a trend towards improved transplant outcomes compared to male recipients in this multi-factorial analysis (8). Given the well-documented changes across the lifespan, it appears probable that sex hormone levels are critical for age-dependent sex differences in transplantation outcomes.

Aging is associated with distinct physiological and pathological changes in almost all organs and organ systems that also affect immunity, a process coined immunosenescence. Immunosenescence, considered a major hallmark of aging, is characterized by chronic low-grade inflammation, often referred to as inflammaging, a process that goes along with a general immune dysfunction (10). Immunosenescence affects innate, and particularly the adaptive immune system. Those alterations produce shifts in both, overall and proportional counts of immune cells, their distribution, proliferation, activation, and efficacy, coupled with an augmented secretion of inflammatory cytokines, proteases, growth factors, and angiogenic factors produced by senescent cells, referred to as the senescence-associated secretory phenotype (SASP) (10–13). Clinically, those alterations manifest through higher rates of severe and lethal infections, augmented cancer rates, increased incidences of certain autoimmune diseases, as well as compromised responses to vaccinations in the elderly (14, 15).

While aging and the associated development of immunosenescence remains currently an inevitable biological phenomenon, its pace and manifestation are subject to several considerable individual intrinsic and extrinsic determinants. Behavioral components including physical inactivity, diets, or substance abuse may lead to chronic inflammatory changes, accelerating immunosenescence (16). Biological sex, in turn, may contribute through co- or independent factors during aging.

Longevity is a recognized sex-dependent phenomenon. Studies consistently indicate that women tend to outlive men (17). This phenomenon can be attributed to a complex interplay of sex- and gender-dependent biological, behavioral, and social factors. However, the underlying reasons for this phenomenon are still not fully understood. While the extent of the sex-based longevity gap varies across different geographic regions, the consistent advantage of the female sex in terms of lifespan underscores the significant role of biological factors (18).

Females and males also show distinct differences in their susceptibility to and the course of specific diseases. Male sex, for example, is associated with a higher risk of developing cancer in all age groups. Additionally, men are more likely to suffer from severe infections and higher mortality rates resulting from sepsis. Conversely, autoimmune diseases including Sjögren's Syndrome and Multiple Sclerosis in addition to neurodegenerative disorders such as Alzheimer's disease occur more often in women compared to men (19). Sex hormones and genetic variations including the presence of two X chromosomes in women, are likely significant contributors to the sex-specific disparities in longevity and morbidity that go along with a wide range of physiological and pathological processes, including sex-specific aspects of immunity and immunosenescence (19–21).

Gender aspects determined by socioeconomic and biographical factors including lifestyle, childbearing and -raising, cultural and work activity related components as known to interfere with sex- and age-dependent differences impacting longevity, health, diseases, and also transplant outcomes. Nevertheless, quantifying these factors remains challenging while effects on medication adherence and overall compliance have been recognized. As such, gender-specific components may influence the clinically observed differences in transplantation outcomes associated with sex and age (22, 23). At the same time, biological sex and gender-specific aspects may differ in regard to their effects on transplant outcomes. Of note, a recent study comparing the adherence in young female vs. male kidney transplant recipients showed that female recipients presented with an improved adherence to immunosuppression (24). An additional study supported those observations, identifying male sex as a risk factor for non-adherence (25). Work by us and others has focused on the effects of biological sex showing augmented acute rejection rates in young female recipients, emphasizing on the relevance of biological sex and age factors affecting transplant outcomes (4, 8).

Here, we summarize data showing the effects of biological sex on transplantation outcomes and alloimmune responses with an emphasis on age-dependent effects. Of note, we define sex as a biological variable, which needs to be distinguished from the term gender, expressing the socio-cultural and psychological identity of a person.

## Clinical relevance of biological sex and aging in solid organ transplantation

Both, donor and recipient age are widely accepted as risk factors for inferior outcomes following SOT reflected by age limits for organ donation and transplantation in addition to specific allocation programs including the Eurotransplant Senior Program (ESP) (26–28).

Despite the substantial body of evidence showing the significance of donor and recipient sex affecting transplantation outcome, biological sex has thus far rarely been considered for clinical decision-making. This approach can be attributed partially to the lack of available consistent data and, for organ allocation, to the overall scarcity of organs. Of additional relevance, most clinical studies examine sex disparities without recognizing age as a significant confounding factor.

### Age and sex in kidney transplantation (KTx)

The analysis of more than 29,000 female and more than 44,000 male KTx recipients in the USA analyzing sex differences for acute rejection and chronic allograft failure in KTx patients has shown that female recipients had an augmented relative risk for acute rejections. Male sex in this study was associated with an increased relative risk for chronic allograft failure, an effect which was shown to be age-dependent and more frequently observed in older recipients (29).

A large volume study of approximately 160,000 KTx recipients listed in the SRTR addressed whether sex-specific differences in graft failure change with age. The study found that female recipients of male donor kidneys had inferior graft survival when compared to male recipients independent of recipient age. In contrast, when transplanting kidneys from female donors, only adolescent and young female recipients presented with inferior outcomes compared to male recipients of comparable age. Of relevance, female recipients ≥45 years receiving a female organ showed significantly improved graft survival when compared to male recipients of the same age (3).

Our own more recent study in more than 400,000 KTx patients listed with the SRTR, transplanted between 1987 and 2017 demonstrated inferior graft survival in young female KTx recipients (15–34 years) when compared either to their male counterparts or to old female recipients (55–74 years) independent of donor sex. This effect was more pronounced when kidneys of male donors were transplanted. Again, consistent with previous findings, graft survival in older recipients (55–74 years) was significantly better in women compared to men, independent of donor sex (8).

Our own results are also supported by those of others who analyzed more than 25,000 kidney transplant recipients in the UK. In this study, the authors report on findings in support of our data with higher rates of death-censored graft loss rates in young female recipients of male kidneys compared to male recipients of similar age. While not reaching statistical significance, the authors note, much like our own observations, that these disparities gradually decreased with advanced recipient age (30).

An analysis of more than 400,000 KTx recipients listed in the SRTR, ANZDATA, and CTS databases confirmed previously published data in young female recipients of male allografts with higher rates of death-censored graft failure rates compared to male recipients of comparable age. Again, this discrepancy was not observed in older recipients (4).

### Age and sex in liver transplantation (LTx)

For LTx, which ranks as the second most transplanted organ worldwide, there is comparatively less available data.

More than 46,000 patients receiving LTx in Europe between 2002 and 2012 showed significantly higher 10-year overall survival in female LTx recipients. Donor as well as recipient age >60 years was associated with inferior outcomes for both sexes. Notably, age-matched transplants demonstrated a reduced risk of unfavorable outcomes for both, male and female recipients (31). Noteworthy, this study did not analyze the impact of different recipient age groups on sex disparities.

More recently, Simone et al. analyzed LTx recipients listed in the SRTR, transplanted between 1988 and 2019. The authors investigated the impact of recipient sex by age group on graft survival and demonstrated significantly improved transplant outcomes in female recipients ≥45 years receiving female livers compared to male recipients of comparable age. Those differences could not be observed in young recipients (<45 years) of female organs. In contrast, young female recipients (<45 years) of male organs presented with higher graft failure rates compared to male recipients of the same age, although this difference did not reach statistical significance (9).

### Age and sex in lung and heart transplantation (luTx and HTx)

Only limited data are available for HTx and LuTx regarding a more detailed analysis of sex and age. A retrospective study of more than 6,000 patients from the United Network for Organ Sharing Standard registry, receiving LuTx in 2015 for idiopathic pulmonary fibrosis showed an increased mortality rate in males compared to female LuTx recipients. This effect was particularly prominent in recipients ≥65 years suggesting an age-dependent impact (5).

A more recent analysis of close to 70,000 HTx recipients demonstrated higher graft failure rates in female recipients of male donor grafts in all age groups except for recipients aged 25–44 years. The most notable disparity in graft failure rates was observed in patients aged 13–24 years. These discrepancies were not evident in patients receiving female grafts, regardless of age (32).

## Relevance of donor-recipient sex and age mismatch

**Figure 1:**
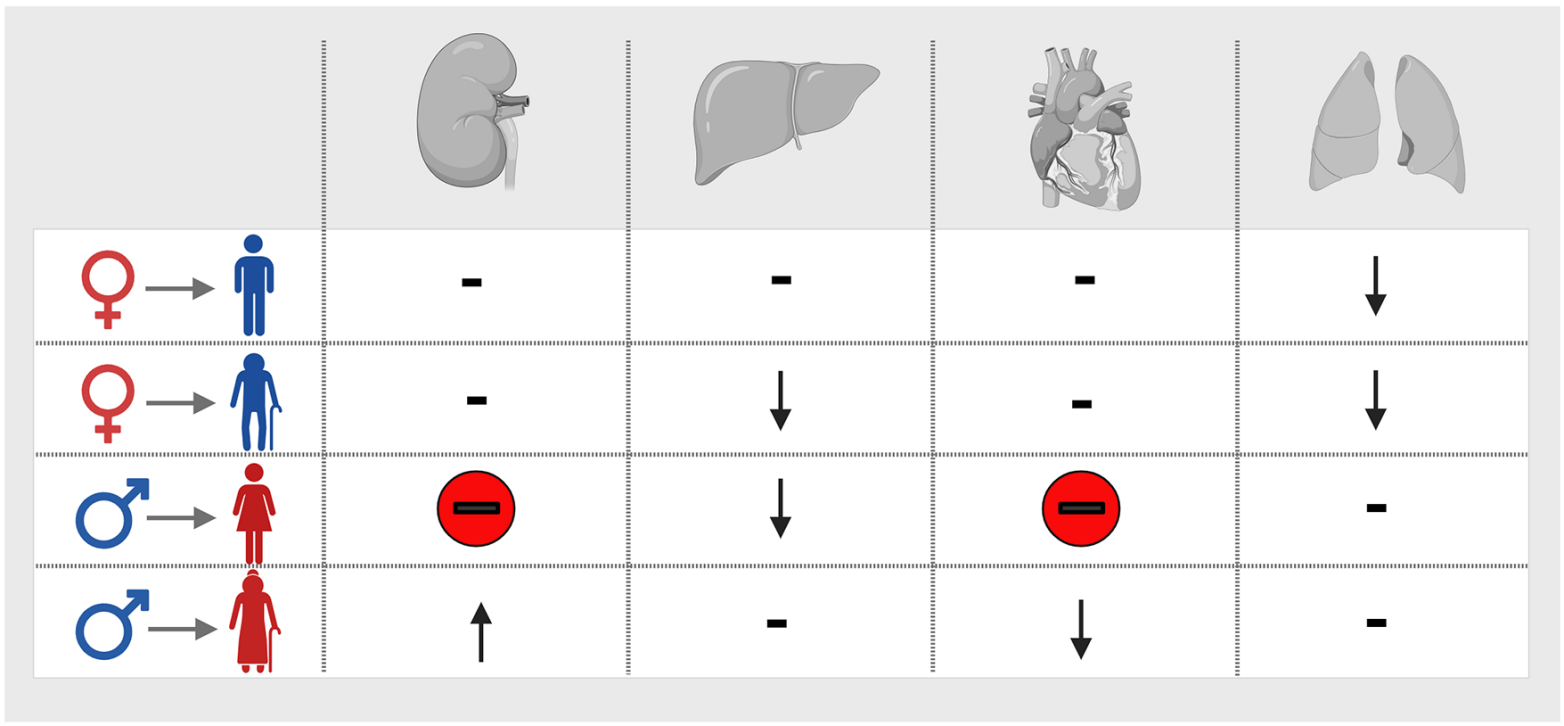
Recipient age affects the relevance of donor-recipient sex mismatches for transplantation outcome in kidney, liver, heart, and lung transplantation. Potential mismatches are represented by the symbols for female and female donors, and old and young male and female recipients in the first column. ↑, favorable outcomes; ↓, unfavorable outcomes; red minus sign, worst combination in comparison with any other age- and sex-group. Of note, data on lung transplantation apply exclusively to patients with idiopathic pulmonary fibrosis (3, 5, 8, 9, 32). Created with BioRender.com.

Sex and age affect organ donors as well as recipients, leading to a multitude of potential mismatches between donors and recipients (Figure 1). Mismatches, in turn, may hold comparable relevance to factors exclusively tied to either recipient or donor.

The relevance of donor-recipient sex mismatches has been reported for different organs, albeit with conflicting results. We have recently analyzed the complexity of sex and sex mismatches in an organ-specific fashion. In brief, outcomes for sex-matched transplantations seem to yield superior outcomes (6, 7). Potential explanations may include size-mismatches in addition to the presence of the H-Y antigen on male organs, representing a minor histocompatibility antigen (33–35).

Potential implications and benefits of age-matched organ allocation continue to be a focus of an ongoing broad discussion. Donor age has been linked to inferior outcomes in heart, lung, kidney, and liver transplantation (36–39). The scarcity of organs, at the same time combined with an increasing number of old organ donors, have resulted in an increased utilization of these organs.

Conceptually, organs from old donors pose a greater immunogenetic challenge, intensifying alloimmunity with higher rates of acute rejection episodes during the early post-transplantation period (40). Furthermore, older organs are more susceptible to injury and less likely to recover after prolonged ischemia or perioperative damages (41, 42). Hence, the less robust alloimmunity in aging may represent an advantage when transplanting older organs into older recipients.

The ESP has put this concept into practice. This model was introduced in 1999 in Europe to address the general organ shortage and the need for older patients, in particular by transplanting kidneys from donors aged ≥65 years into recipients aged ≥65 years without prospective HLA-matching. Long-term follow-up (20 years) showed overall favorable short- and long-term outcomes for patients undergoing KTx within the ESP, promoting the advantages of age-matched transplantations. Nonetheless, recipients in the ESP experienced higher rates of acute rejections with T-cell-mediated rejection episodes emerging as a distinct risk factor for graft failure and mortality (28, 43). Those data put into perspective the ESP HLA-matching policy as well as the hypothesis of the potential benefits of an aged immune system in the transplant setting. Of additional relevance, ESP data also showed male recipient sex as a risk factor for increased mortality (28).

Notably, studies investigating the relevance of donor-to-recipient age mismatches and the potential relevance of the donor-recipient age gap have been inconsistent. A study from 2019 analyzing more than 25,000 HTx between 2005 and 2018 registered in the United Network for Organ Sharing (UNOS) database could not show an effect of donor-recipient age mismatch on transplantation outcome (36). A single-center study of 409 LuTx patients came to the same conclusion (44).

In contrast, a more recent study, analyzing UNOS data from more than 28,000 HTx patients revealed that receiving an organ from a donor >5 years older was associated with adverse outcomes. In contrast, receiving an organ from a donor >5 years younger led to favorable outcomes. Most interestingly the favorable effects of receiving a young organ diminished with increasing recipient age (45). In more than 63,000 LTx patients in the OPTN/UNOS database transplanted between 2002 and 2015, it has been shown that receiving organs from donors aged >40 years was associated with adverse transplantation outcomes only in recipients younger than 40 years but not when those organs had been transplanted into recipients >60 years of age (46).

Taken together, sex and age donor-recipient mismatches affect transplant outcomes in an organ-specific fashion. The combinatorial effect of sex and age mismatch has been studied only by few so far, thus warranting more detailed clinical organ-specific studies. Nevertheless, our data and those of others emphasize on the relevance of donor-recipient sex and age mismatches. The finding, that particularly young female recipients of male organs present with adverse outcomes, further hints to a combinatorial effect of sex and age for recipient related as well as donor related factors (8, 33). Age-specific sex hormone levels, the development of immunosenescence with age, together with age-specific pharmacokinetics and -dynamics, and medication adherence depending on age and sex in addition to organ-specific effects may be of critical relevance.

## Sex hormones, aging and alloimmunity

**Figure 2:**
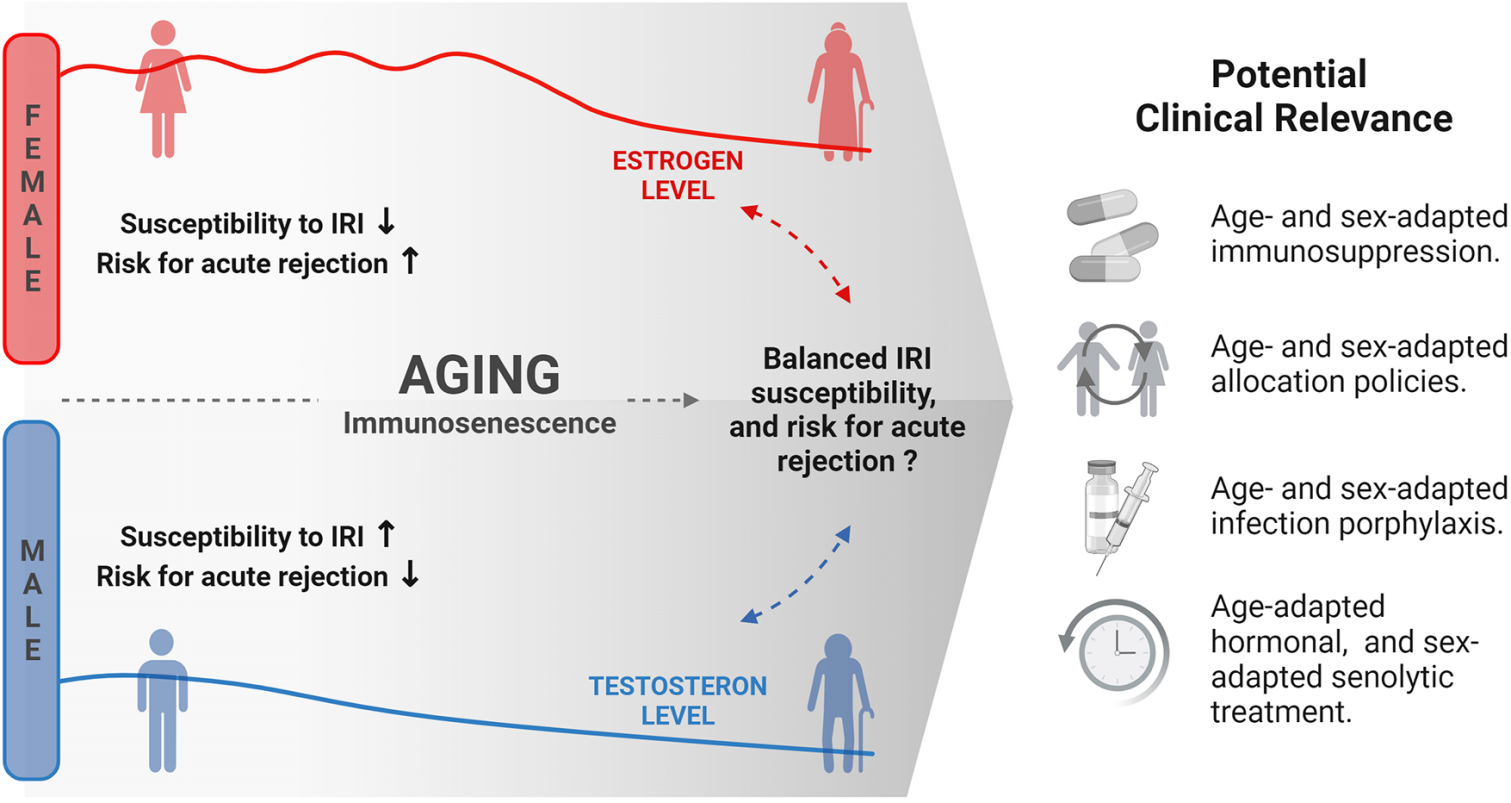
Changing sex hormone levels in females and males over lifetime and its potential clinical relevance for transplantation outcome and clinical decision making. IRI, ischemia reperfusion injury. Created with BioRender.com.

Changing levels of sex hormones over a lifetime seem relevant for the observed age and sex-related differences in transplantation outcomes (Figure 2).

Sex hormone levels change in age-specific ways affecting estrogen levels with particular relevance in females. While estrogen levels are low during childhood, puberty is accompanied by a steep increase with high levels in reproductive age and even higher levels during pregnancy. However, levels drastically decrease during menopause, ultimately leading to low levels in postmenopausal women. In contrast, testosterone levels in men tend to remain relatively stable, showing only a slow decrease in aging after an initial rise during puberty (47).

Sex hormones exert significant influences on diverse physiological functions thereby shaping the course of various diseases such as cardiovascular diseases, infectious diseases, and autoimmune disorders (19, 20, 48, 49). Estrogens have been observed to modulate the function of numerous innate and adaptive immune cells in a dose-dependent manner. Effects are mediated by “classical” estrogen receptors (ER), including ERα and ERβ that are expressed on a variety of immune cells such as monocytes, DCs, T cells and B cells. Membrane-associated receptors, G-protein coupled receptors and even receptor-independent pathways play additional roles in exerting estrogen-specific effects. Via those mechanisms, estrogens modulate gene expression and non-genomic pathways which are pertinent to various functions, including (allo)immunity (7, 50).

Studies investigating the role of sex hormones for alloimmunity have been limited thus far. We have recently shown that ovariectomies and thus estrogen deprivation in young female mice prolonged allograft survival. Graft survival in old female mice, in turn, was comparable to those in ovariectomized or young naïve controls. We were also able to detail the effects of estrogen levels on T-cell proliferation and differentiation after allotransplantation and demonstrated that estrogen deprivation attenuated CD4+ T cell responses. Furthermore, high estrogen levels, comparable to those during reproductive age in women, promoted pro-inflammatory Th1 and Th17 activation and proliferation (8).

While studies delineating the effects of age and biological sex in transplantation have been limited thus far, several studies have emphasized on the substantial impact of sex hormones in immunity thus stressing the relevance of sex-hormones in alloimmunity.

## Aging and biological sex affecting ischemia reperfusion injury (IRI)

The consequences of IRI affect outcomes after SOT broadly (51). Female recipient sex mainly attributed to the influence of estrogens has been shown to protect against IRI (52–54). A variety of pre-clinical studies in kidney, heart, and livers has shown a protective role of estrogens against IRI (55–59). Underlying pathways, although only incompletely understood include the influence of estrogens on mitochondrial activity, the expression of antioxidant enzymes, and inflammatory responses subsequent to IRI (58, 60, 61).

Conversely, advanced organ age increases susceptibility towards IRI (62–64) with mitochondrial dysfunction, increased oxidative stress, impaired autophagy, endothelial dysfunction, and an overall chronic-proinflammatory state playing an important role in increasing tissue vulnerability, exacerbates IRI and alloimmunity (63). The accumulation of SCs in aging furthermore alters resilience towards IRI, reducing regenerative capacities (65).

Overall, the interplay between sex and age in IRI appears complex. In young females, estrogens play a predominantly protective role, ameliorating the consequences of IRI while the protective effects of estrogens may be less pronounced with aging (54, 66). In support, pre-clinical studies investigating the effects of selective estrogen receptor modulators and treatment suggest that the loss of protection in aging could at least in part be preserved (55, 67).

## Conclusion and further considerations

Existing clinical and pre-clinical data strongly suggest that age plays a relevant role in influencing sex-related variations in alloimmune responses and transplant outcomes. Yet, clinical evidence and our understanding of underlying mechanisms remain limited. Ageing is an inter-individual varying process and as such complex to analyze. Chronological and biological age may differ widely depending on a variety of influencing factors including comorbidities, lifestyle, and biological sex. Epigenetic clocks might provide a possible avenue to measure biological age in donors and recipients, aiding in organ allocation and the fine-tuning of immunosuppression.

Furthermore, the relevance and drivers of sex-dependent components driving age-related diseases including cancer and infections with relevance to transplantation compounded by the effects of immunosuppression remain understudied. Indeed, there may be a relevant rationale for sex and age-adapted immunosuppression and infection prophylaxis.

Sex hormone levels change over a lifetime and affect (allo-)immune responses (21). The modulation of sex hormone levels may thus provide a potential therapeutic target for further individualized sex- and age-adapted treatment of transplantation recipients. The relevance of changing estrogen receptor expression and function may represent relevance for potential therapeutic targets (68). Notably, other sex-affected processes occurring with age including alterations in X-chromosome skewing with age might play an additional role in sex-specific changes in immune response changes with relevance for alloimmune response (69).

In general, greater emphasis should be placed on considering age as a pertinent factor when examining sex disparities, with relevance in and beyond transplantation. Moreover, establishing a more standardized definition of “old” and “young” may improve the consistency of clinical data and facilitate future research. Correlating those definitions with sex-related differences in aging will require attention moving forward.

In summary, both sex and age play an important and complex role in transplantation. The complex interplay includes both, donor and recipient factors, hormonal, organ-specific and genetic components that will require further detailed research.
